# Colonoscopic surveillance improves survival after colorectal cancer diagnosis in inflammatory bowel disease

**DOI:** 10.1038/sj.bjc.6605359

**Published:** 2009-10-13

**Authors:** M W M D Lutgens, B Oldenburg, P D Siersema, A A van Bodegraven, G Dijkstra, D W Hommes, D J de Jong, P C F Stokkers, C J van der Woude, F P Vleggaar

**Affiliations:** 1 Department of Gastroenterology and Hepatology, University Medical Center, Utrecht, The Netherlands; 2 Department of Gastroenterology and Hepatology, VU University Medical Center, Amsterdam, The Netherlands; 3 Department of Gastroenterology and Hepatology, University Medical Center Groningen, Groningen, The Netherlands; 4 Department of Gastroenterology and Hepatology, Leiden University Medical Center, Leiden, The Netherlands; 5 Department of Gastroenterology and Hepatology, Radboud University Nijmegen Medical Center, Nijmegen, The Netherlands; 6 Department of Gastroenterology and Hepatology, Academic Medical Center, University of Amsterdam, Amsterdam, The Netherlands; 7 Department of Gastroenterology and Hepatology, Erasmus Medical Center, Rotterdam, The Netherlands

**Keywords:** inflammatory bowel disease, colorectal cancer, surveillance, survival, tumour stage, IBD, CRC

## Abstract

**Background:**

Colonoscopic surveillance provides the best practical means for preventing colorectal cancer (CRC) in inflammatory bowel disease (IBD) patients. Strong evidence for improved survival from surveillance programmes is sparse.

**Method:**

The aim of this study was to compare tumour stage and survival of IBD patients with CRC who were a part of a surveillance programme with those who were not. A nationwide pathology database (PALGA (pathologisch anatomisch landelijk geautomatiseerd archief)) was consulted to identify IBD patients with CRC treated in all eight university hospitals in The Netherlands over a period of 15 years. Patients were assigned to the surveillance group when they had undergone one or more surveillance colonoscopies before a diagnosis of CRC. Patients who had not undergone surveillance served as controls. Tumour stage and survival were compared between the two groups.

**Results:**

A total of 149 patients with IBD-associated CRC were identified. Twenty-three had had colonoscopic surveillance before CRC was discovered. The 5-year CRC-related survival rate of patients in the surveillance group was 100% compared with 74% in the non-surveillance group (*P*=0.042). In the surveillance group, only one patient died as a consequence of CRC compared with 29 patients in the control group (*P*=0.047). In addition, more early tumour stages were found in the surveillance group (*P*=0.004).

**Conclusions:**

These results provide evidence for improved survival from colonoscopic surveillance in IBD patients by detecting CRC at a more favourable tumour stage.

The increased risk of colorectal cancer (CRC) in patients with inflammatory bowel disease (IBD) has been well documented (Eaden *et al*, 2001; Jess *et al*, 2005). In an effort to detect dysplasia or early stage cancer, it is advised that patients should enter a surveillance programme according to the guidelines of the American Gastroenterological Association (Winawer *et al*, 2003) or of the British Society of Gastroenterology (Eaden and Mayberry, 2002). Although not flawless, it is currently the best tool available to detect early-stage neoplasia or prevent CRC in patients with IBD. The level of evidence for its effectiveness, however, is low and has only been studied in patients with ulcerative colitis (Collins *et al*, 2006). The ideal setup to evaluate the effectiveness of surveillance programmes would be a randomised trial with CRC-related mortality as the primary end point. Unfortunately, such a trial does not exist and probably never will be performed because of practical and ethical considerations (Bernstein, 1998). Therefore, retrospective studies are probably the best alternative to assess the effectiveness of colonoscopic surveillance.

A recent review by the Cochrane Collaboration (Collins *et al*, 2006) identified three studies that presented indirect evidence for improved survival from colonoscopic surveillance in ulcerative colitis patients (Lashner *et al*, 1990; Choi *et al*, 1993; Karlen *et al*, 1998). These studies either had a low number of patients with IBD-associated CRC or a low number of patients in a surveillance programme, which prevented the outcomes from reaching statistical significance.

We recently reported on time intervals between the diagnosis of IBD and CRC in a large cohort of patients with IBD-associated CRC, collected from all university medical centres in The Netherlands using a nationwide pathology database (Lutgens *et al*, 2008). The aim of this retrospective study was to compare tumour stage and survival in this cohort between patients who were a part of a surveillance programme with those who were not.

## Materials and methods

### Search strategy

Pathologisch anatomisch landelijk geautomatiseerd archief (PALGA), the nationwide network and registry of histo- and cytopathology (Casparie *et al*, 2007), contains pathology reports generated in the Netherlands dating back to 1971. These reports are concluded with diagnostic terms in line with SNOMED (systematised nomenclature of medicine) terminology and were used to search for patients with IBD-associated CRC. PALGA achieved nationwide coverage in 1990. For that reason, a search for the time period between January 1990 and July 2006 in all eight Dutch university medical centres for synchronous or metachronous diagnoses of IBD and CRC was conducted. The following combinations of search terms were used: ulcerative colitis and adenocarcinoma; Crohn's disease and adenocarcinoma; colon and colitis and adenocarcinoma; colon and inflammation and adenocarcinoma; colon and chronic inflammation and adenocarcinoma; colon and idiopathic colitis and adenocarcinoma; colon and adenocarcinoma and active inflammation. Only patients with a confirmed histological diagnosis of inflammatory bowel disease and CRC were included.

The PALGA search engine ensures patient privacy by supplying anonymous data. Only the patient's own physician was able to link PALGA identification codes to real patient identification codes. The treating physician in each university medical centre subsequently supplied only the first author with anonymous data. No personal patient data were recorded.

### Data extraction

The following data were collected from patient charts by one investigator (ML): type of IBD, gender, age at IBD diagnosis, age at CRC diagnosis, date of onset of symptoms, date of IBD diagnosis, date of CRC diagnosis, date of start of surveillance, intervals between surveillance colonoscopies, date of end of follow-up, cause of death, tumour stage in the resection specimen or by radiological imaging if the patient was not operated, concurrent primary sclerosing cholangitis, existing co-morbidity, history of smoking and alcohol use, and history of 5-amino-salycilic acid (5-ASA) medication. A diagnosis of primary sclerosing cholangitis had to be made with retrograde cholangiopancreatography, magnetic resonance cholangiopancreatography, or liver biopsy. Co-morbidity was defined as severe cardiac, severe pulmonary, severe renal, severe liver dysfunction, or malignancy other than CRC.

### Surveillance criterion

A patient was assigned to the surveillance group when before CRC diagnosis at least one or more surveillance colonoscopies at regular intervals (every 1–3 years) had been performed. The remainder of the patients were assigned to the non-surveillance group and served as controls. The surveillance quality had to meet the standard that is described by current guidelines (Eaden and Mayberry, 2002; Winawer *et al*, 2003). This entails the intent to detect neoplasia by taking four random biopsies every 10 cm of the colon in addition to targeted biopsies of suspicious areas during that colonoscopy. No attempt was made to compare surveillance colonoscopies with each other on the basis of frequency or number of biopsies.

Patients who were under regular surveillance for multiple years, but for some reason had skipped one colonoscopy, were only assigned to the surveillance group if the longest lapse since the last colonoscopy was 3 years or less. To minimise selection bias, patients in whom the diagnosis of CRC was made by colonoscopy according to surveillance protocol for the first time (thus not yet enrolled in a surveillance programme) were not assigned to the surveillance group when the reason for this first surveillance colonoscopy was new or recurrent symptoms of disease.

### Statistical analysis

The χ^2^ test, Fisher's exact test, and Student's *t*–test were used where appropriate to compare patient characteristics between the two groups. Kaplan–Meier (Kaplan and Meier, 1958) and Cox regression analyses were used for survival calculations. The primary study end points were CRC related or overall death. The end of follow-up was either the end of study date (1 July 2006) or date of death. When a patient was lost to follow-up, the date of the last visit to the hospital was recorded as end of follow-up. The Tarone–Ware test of equality of survival distributions was used to compare differences between survival curves. Tumour stages were grouped according to the sixth edition staging system of the American Joint Committee on Cancer (Greene *et al*, 2002; Sobin and Wittekind, 2002). The χ^2^ test and Fisher's exact test were used where it was appropriate to compare tumour stages between the surveillance and non-surveillance groups. *P*-values <0.05 were considered to be statistically significant. SPSS software for windows version 14.0, SPSS Inc, was used for all statistical analyses.

## Results

Our search identified 166 patients, of whom 17 were excluded. The reasons for exclusion were: no histological confirmed diagnosis of IBD (*n*=11), suspected adenocarcinoma in the biopsy sample that could not be reproduced in the colectomy specimen (*n*=2), a focus of micro-carcinoid instead of adenocarcinoma (*n*=2), unknown date of IBD diagnosis (*n*=1), and occurrence of CRC before IBD was diagnosed (*n*=1). This left 149 cases for analysis. Twenty-three patients had undergone one or more surveillance colonoscopies before the diagnosis of CRC and were analysed as the surveillance group. Surveillance was started after a median of 14.3 (std 8.0) years after histological diagnosis of IBD. CRC developed after a median of 6.4 years (range 1–21) after initiation of surveillance. The remaining 126 patients were assigned to the non-surveillance control group. Patient characteristics are shown in Table 1. No statistically significant differences were found between the two groups for any of the variables.

Follow-up after a diagnosis of CRC was complete for 114 (79%) patients until the study end date or death. Thirty-one (21%) patients were lost to follow-up. Four of these 31 patients, two in the surveillance group and two in the non-surveillance group, were lost to follow-up immediately after diagnosis of CRC and were therefore excluded from the survival analyses.

**Figure 1:**
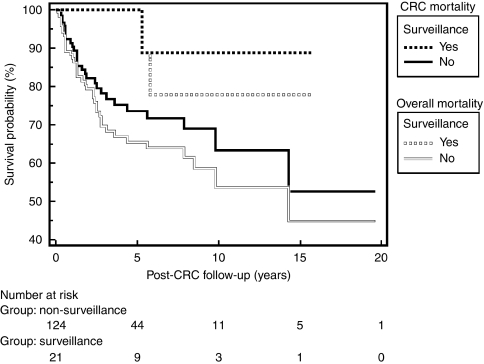
Survival analysis for CRC-related and overall mortality. The Tarone–Ware test of equality of survival distribution was used to compare survival between the two groups. CRC-related mortality is presented by solid squares and line. Overall mortality is presented by outlined squares and line. For CRC-related mortality, the 5-year survival in the surveillance group (solid squares) was 100% compared with 74% in the non-surveillance group (solid black line) (*P*=0.029); the primary end point was date of death due to CRC; cases were censored for date of end of study, date of death related to any other primary malignancy, date of death related to colectomy, date of death by any other cause, and date of lost to follow-up. For overall mortality, the 5-year survival in the surveillance group (outlined squares) was 100% compared with 65% in the non-surveillance group (outlined line; *P*=0.042); the primary end point was date of death; cases were censored for date of end of study period and date of lost to follow-up. CRC, colorectal cancer.

Overall, 42 of 145 (29%) patients died. The cause of death was directly related to metastasised CRC in 30 patients. Of the remaining, six patients died from metastasis of a different primary tumour (cholangiocarcinoma (*n*=3), renal cell tumour (*n*=1), urothelial carcinoma (*n*=1), primary tumour of the stomach (*n*=1)), and another six patients died after complications related to colectomy. In the surveillance group, only one patient died because of CRC compared with 29 patients in the non-surveillance control group (*P*=0.047). The overall 5-year survival rates in the surveillance group and in the non-surveillance control group were 100% and 65%, respectively (*P*=0.029) (Figure 1). CRC-related 5-year mortalities were 0% and 26% in the surveillance and non-surveillance groups, respectively (*P*=0.042) (Figure 1). In addition, a multivariate Cox regression analysis, including type of IBD, concurrent primary sclerosing cholangitis, age at CRC diagnosis, and co-morbidity as co-variables confirmed the association between colonoscopic surveillance and improved survival with reduced CRC-related mortality; however, this did not reach statistical significance (*P*=0.10) (Table 2).

Eleven patients in the non-surveillance group were diagnosed with IBD and CRC simultaneously. Exclusion from analysis of these patients strengthens the effect. The 5-year overall mortality is 0% in the surveillance group and 36% in the non-surveillance group (*P*=0.02). For CRC-related mortality, these percentages are 0% and 29% (*P*=0.03). This effect also remains visible in the multivariate analysis, although again it is not statistically significant (Table 2).

In 10 out of 149 patients (7%), we did not have information regarding 5-ASA prescription. This leaves 139 patients for analysis, of whom 119 (86%) have used a 5-ASA preparation during the course of their disease. Of these 119 patients, 64 (54%) used 5-ASA medication for more than three-quarters of their disease duration. Nevertheless, all these patients developed CRC despite the chemopreventive nature of 5-ASAs. As mentioned earlier, 4 out of 149 patients were excluded when evaluating survival because they were lost to follow-up directly after CRC diagnosis. Therefore, 135 patients remain for 5-ASA analysis between groups. Twenty patients were in the surveillance group and 115 in the non-surveillance group. All 20 patients (100%) in the surveillance group had used 5-ASA preparations during their disease. In the non-surveillance group, 96 out of 115 (77%) had used 5-ASA preparations. This difference showed a trend but was not statistically significant (*P*=0.08). Furthermore, when included in the multivariate cox regression analysis, it has no influence on survival (*P*=0.96), whereas the effect of surveillance (*P*=0.098) remains unchanged.

Tumour stages of the two groups are separately shown in Table 3. Tumours were staged by a pathologist on the resection specimen in 138 patients (93%) according to the TNM classification. Information on TNM stage could not be retrieved in 11 patients (7%) for the following reasons: either the patients were not operated on because of metastatic disease (*n*=6), or a fully detailed pathology report of resection specimen was not retrievable (*n*=5). In patients from the surveillance group, the observed number of lower-stage tumours was higher than the expected number. This effect was statistically significant when Stage 0 and 1 tumours were compared with all higher stages between the two groups. In the surveillance group, 12 patients (52%) had Stage 0 and 1 tumours compared with 28 patients (24%) in the non-surveillance group (*P*=0.004). Moreover, the same effect was also observed in the opposite direction with a statistically significantly lower number of patients with Stages 3B-C and 4 tumours: four patients (17%) in the surveillance group compared with 48 patients (42%) in the non-surveillance group (*P*=0.049).

Despite surveillance, four cancers in the surveillance group were diagnosed as a consequence of new or altered symptoms of disease. These so-called interval cancers had varying tumour stages: T1N0M0, T2N1M0, T3N0M0, and T4N0M0. The interval since last surveillance colonoscopy was 10, 14, 8, and 7 months, respectively, and the patients were on 1-, 2-, 1, and 3-yearly intervals of surveillance colonoscopies.

## Discussion

This series shows an improved survival in IBD patients who developed CRC and in whom colonoscopic surveillance was performed. It provides evidence showing the efficacy of surveillance on CRC-related mortality with sufficient numbers of patients with IBD-associated CRC. CRC-related mortality was significantly lower in the surveillance group, with a 5-year survival rate of 100% in the surveillance group and 74% in the non-surveillance group. This effect can be explained by the detection of earlier-stage CRC in the surveillance group, which translates into a better prognosis.

This report adds to the relatively small body of evidence, indicating survival benefit through a surveillance strategy in ulcerative colitis. We feel that it is prudent to join the ulcerative colitis and Crohn's colitis data, because there is increasing evidence that the pathogenesis and natural behaviour of inflammation-associated dysplasia in ulcerative colitis and Crohn's disease do not differ, and the risk of CRC is increased in both (1,2). A recent systematic review (Collins *et al*, 2006) by the Cochrane collaboration detected only three papers (Lashner *et al*, 1990; Choi *et al*, 1993; Karlen *et al*, 1998) that properly addressed the question of surveillance effectiveness for patients with ulcerative colitis. Other surveillance studies lacked valid control groups (Rosenstock *et al*, 1985; Lofberg *et al*, 1990; Nugent *et al*, 1991; Jonsson *et al*, 1994; Friedman *et al*, 2001; Biasco *et al*, 2002; Hata *et al*, 2003) or were not designed to answer this question (Eaden *et al*, 2000). The reviewers of the Cochrane paper concluded that all three publications pointed towards a beneficial effect of surveillance on survival, but that the evidence was indirect. Lashner *et al* (1990) showed an improved overall survival in the surveillance group, but were unable to show that this was secondary to a reduced CRC-related mortality. Similarly, they could not find a difference in tumour stage between the two groups. Choi *et al* (1993) published a small series of 41 patients who developed CRC associated with ulcerative colitis, of whom 19 were in a colonoscopic surveillance programme and 22 were not. In their series, as in ours, CRC was detected at a significantly earlier stage in patients who had undergone colonoscopic surveillance. Karlen *et al*, (1998) identified a trend towards a protective effect of colonoscopic surveillance.

The strength of the current series lies in the high number of cases with IBD-associated CRC available for analysis and the strict criteria for inclusion in the surveillance group. Moreover, no differences between the two groups in age at cancer diagnosis, length of follow-up, and interval between onset of IBD and CRC diagnosis were found, eliminating confounding from differences in these variables (Table 1). The multivariate analysis confirmed the association between colonocopic surveillance and improved survival, but failed to reach statistical significance most probably because of a type II error resulting from multiple variables in the analysis (Table 2). The putative additional effect of smoking or alcohol could not be evaluated in this analysis because of high percentages of missing values.

Selection is a potential bias that needs consideration. Patients who presented with diarrhoea or rectal bleeding and were subsequently diagnosed with both IBD and CRC could very well have affected the observed results. These patients would not have participated in an endoscopic surveillance programme and were probably only detected with IBD because of their CRC-related symptoms. Eleven patients in our series were simultaneously diagnosed with IBD and CRC. However, our results for CRC-related tumour stage and mortality between groups did not change when patients whose interval between diagnosis of IBD and CRC was less than 1 year were excluded from the analysis. This is not surprising as these patients were probably not different from the patient group that did not undergo surveillance. It seems likely that these individuals did not seek medical care for their condition because of the absence of symptoms, and presented some years after the start of the disease because of symptoms that were found to be (at least partly) CRC related.

It is well recognised that 5-ASA is thought to be chemopreventive for neoplasia. We collected data on medication use in all patients. However, the retrospective design of our data collection warrants us to be careful with its interpretation. Not all physicians meticulously registered the exact duration of medication usage, nor did we have insight into medication adherence.

Lead-time bias is known to influence screening and surveillance data (Kramer, 2004). However, lead-time bias only occurs if the intervention (in this case earlier tumour detection) does not affect the terminal event (in this case mortality by CRC). In the case of CRC, lead-time bias is probably not a significant problem, as earlier diagnosis of CRC at a more favourable stage does improve survival in a beneficial manner (Ries *et al*, 2007; Levin *et al*, 2008). Others have questioned the importance of lead-time bias in CRC-screening studies as well (Ransohoff and Lang, 1991).

Because of the lack of randomisation, volunteer bias might have influenced results. The patients in the surveillance group could have been more health conscious, leading to an earlier diagnosis of CRC. In our study, however, the mean duration of disease until CRC detection was longer in the surveillance group (22.7 *vs* 19.3 years). Therefore, it is not very likely that volunteer bias had a major role in this study.

Four cancers in the surveillance group were found to be interval cancers. This has been observed by others as well (Choi *et al*, 1993). We did not extract information on the exact number of biopsies for each surveillance colonoscopy separately and therefore cannot comment upon whether these interval cancers may be attributable to sub-optimal practice during the previous surveillance colonoscopy. The occurrence of altered bowel symptoms may lead to a diagnosis of CRC. Whether interval cancers are due to a failure of detection during previous colonoscopy or to a rapid progression of cancer is difficult to determine. Back-to-back colonoscopy in patients without IBD has shown that the miss rate for adenomas is 6–27%, which, among others, depends on adenoma size (Rex *et al*, 1997). In addition, in all four of these patients, unifocal low-grade dysplasia (LGD) had been diagnosed before the diagnosis of CRC in a part of the colon close to the location at which subsequently CRC was detected. Therefore, either the CRC was missed at the previous colonoscopy or LGD had progressed towards CRC between the two colonoscopies. Most clinicians recommend colectomy for high-grade dysplasia; however, management for LGD is controversial. Only 5% of Dutch gastroenterologists recommend colectomy for unifocal LGD. A little under 30% of Dutch gastroenterologists recommend colectomy in the case of multi-focal LGD (van Rijn *et al*, 2009).

Finally, referral centre bias deserves attention. All included cases in this study were primarily treated in or referred to tertiary university medical centres, which may have led to the introduction of bias towards patients with more severe disease. Regarding the level of risk of CRC in IBD patients, this kind of bias might be of major importance, but it is less clear how this may affect the main outcome of this study as index and control patients all originate from referral hospitals. Although it is true that the results from this study cannot be translated to the general IBD population unequivocally, to date, no study exists comparing survival outcome between IBD patients from referral centres and population-based cohorts undergoing surveillance.

In conclusion, the patients in the surveillance group showed statistically significant lower CRC-related mortality and more favourable tumour stages. The evidence for improved survival in this paper strengthens the notion that colonoscopic surveillance in patients with longstanding IBD is beneficial and should be performed in patients with a presumed high-risk profile.

## Figures and Tables

**Table 1:** Patient characteristics

	**Surveillance group *N*=23**	**Non-surveillance group *N*=126**	***P*-value**
*IBD*			
Ulcerative colitis	18 (78%)	71 (56%)	0.053
Crohn's disease	5 (22%)	54 (43%)	
Indeterminate colitis	0 (0%)	1 (1%)	
			
*Gender*			
Male	17 (74%)	72 (57%)	0.132
Female	6 (26%)	54 (43%)	
			
Co-morbidity	3 (13%)	29 (23%)	0.410
Median age at IBD-diagnosis (years)	26 (9–50)	30 (6–83)	0.148
Median age at CRC-diagnosis (years)	48 (38–71)	49 (21–85)	0.986
PSC	2 (9%)	17 (14%)	0.739
Mean interval between onset of IBD symptoms and diagnosis of CRC (months)	273 (15–541)	231 (0–536)	0.143
Mean follow-up time after CRC (months)	57 (0–188)^*^	51 (0–235)^*^	0.635

Abbreviations: CRC=colorectal cancer; IBD=inflammatory bowel disease; PSC=primary sclerosing cholangitis.

^*^Four patients were lost to follow-up immediately after diagnosis of CRC and were not included in survival analyses.

**Table 2:** Multivariate cox regression analysis: mortality due to CRC

**Variable**	***P*-value**	***P*-value** a
Type of IBD	0.80	0.74
Age at CRC diagnosis (years)	0.55	0.71
Co-morbidity	0.74	0.64
Primary sclerosing cholangitis	0.16	0.10
Surveillance	0.10	0.08

Abbreviations: CRC=colorectal cancer; IBD=inflammatory bowel disease.

a Exclusion of 11 patients with simultaneous diagnoses of IBD and CRC.

**Table 3:** Tumour stages

**AJCC tumour stage**		**Non-surveillance *N*=121**	**Surveillance *N*=23**	***P*-value**
0	T *in situ*	9	2	0.689
1	T1, T2, N0, M0	19	10	0.008
2A	T3, N0, M0	40	4	0.135
2B	T4, N0, M0	2	1	0.409
3A	T1, T2, N1, M0	3	2	0.180
3B	T3, T4, N1, M0	14	2	1.000
3C	Any T, N2, M0	12	0	0.215
4	M1	22	2	0.367

Abbreviation: AJCC=American Joint Committee on Cancer.

AJCC staging system 6th edition (12;13).

Five tumours were not classifiable under AJCC Staging: 2 TxN0M0, 2 TxNxMx, and 1 TxNxM0.
